# Supplementary material for: Narcissus Plants: A Melting Pot of Potyviruses
Source: Viruses. 2022 Mar 11;14(3):582. doi: 10.3390/v14030582 (PMC8949890; doi:10.3390/v14030582)
Supplement: Supplementary file 1 [file viruses-14-00582-s001.zip › viruses-1597356-supplementary.pdf]

Supplementary Materials

Narcissus Plants: A Melting Pot of Potyviruses

Wiwit Probowati [1,2], Shusuke Kawakubo [1] and Kazusato Ohshima [1,2,*]

[1] Laboratory of Plant Virology, Department of Biological Resource Science, Faculty of Agriculture, Saga University, 1-banchi, Honjo-machi, Saga, Saga 840-8502, Japan

[2] The United Graduate School of Agricultural Sciences, Kagoshima University, 1-21-24 Korimoto, Kagoshima, Kagoshima 890-0065, Japan

* Corresponding author; ohshimak@cc.saga-u.ac.jp

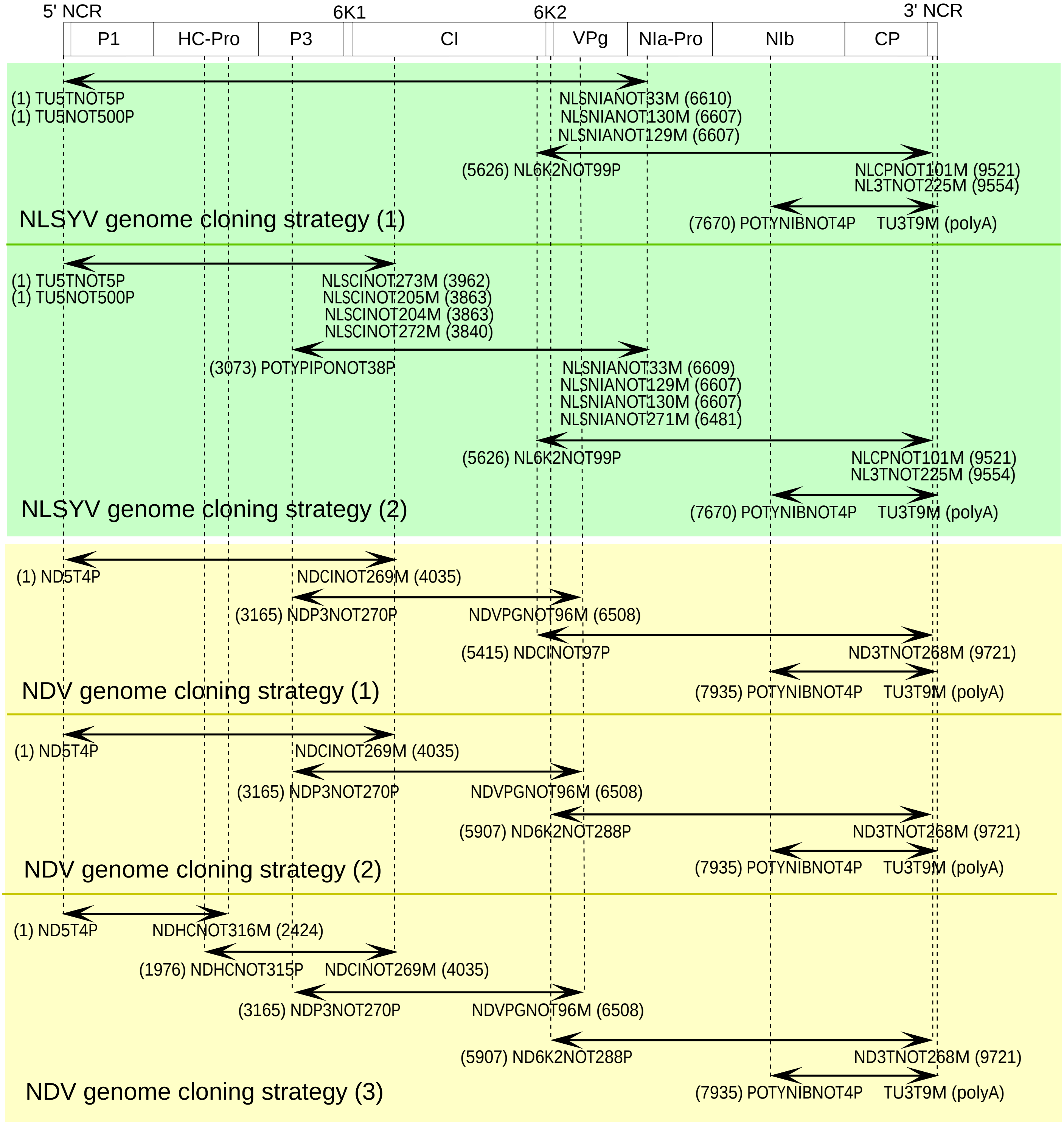

Figure S1. Workflow for full genomic sequencing of narcissus late season yellows virus (NLSYV) and narcissus degeneration virus (NDV). Black lines with arrow heads show the amplified regions by RT-PCR for cloning for all isolates. Numbers in parenthesis correspond to the genomic positions of Zhangzhou isolates of narcissus late season yellows virus [26] and narcissus degeneration virus [24]. NCR; non-coding region, P1; first protein, HC-Pro; helper-component proteinase protein, P3; third protein, 6K1; first 6kDa protein, CI; cylindrical inclusion protein, 6K2; second 6kDa protein, VPg; genome-linked viral protein, NIa-Pro; nuclear inclusion a proteinase protein, NIb; nuclear inclusion b protein, and CP; coat protein.

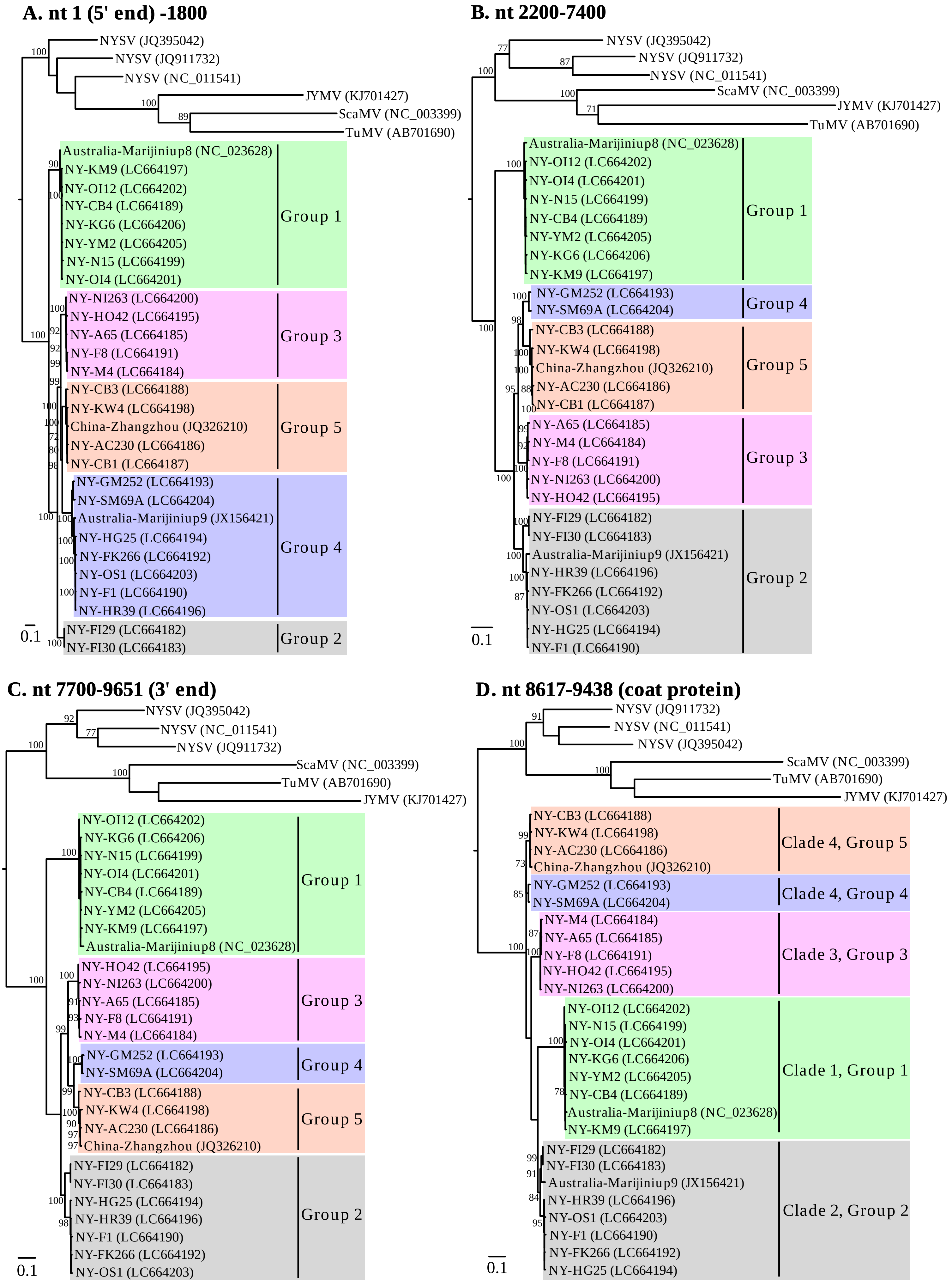
A. nt 1 (5' end) -1800
NYSV (JQ395042)
NYSV (JQ911732)
NYSV (NC_011541)
JYMV (KJ701427)
ScaMV (NC_003399)
TuMV (AB701690)
Australia-Marijiniup8 (NC_023628)
NY-KM9 (LC664197)
NY-OI12 (LC664202)
NY-CB4 (LC664189)
NY-KG6 (LC664206)
NY-YM2 (LC664205)
NY-N15 (LC664199)
NY-OI4 (LC664201)
Group 1
NY-NI263 (LC664200)
NY-HO42 (LC664195)
NY-A65 (LC664185)
NY-F8 (LC664191)
NY-M4 (LC664184)
Group 3
NY-CB3 (LC664188)
NY-KW4 (LC664198)
China-Zhangzhou (JQ326210)
NY-AC230 (LC664186)
NY-CB1 (LC664187)
Group 5
NY-GM252 (LC664193)
NY-SM69A (LC664204)
Australia-Marijiniup9 (JX156421)
NY-HG25 (LC664194)
NY-FK266 (LC664192)
NY-OS1 (LC664203)
NY-F1 (LC664190)
NY-HR39 (LC664196)
Group 4
NY-FI29 (LC664182)
NY-FI30 (LC664183)
Group 2
0.1
B. nt 2200-7400
NYSV (JQ395042)
NYSV (JQ911732)
NYSV (NC_011541)
ScaMV (NC_003399)
JYMV (KJ701427)
TuMV (AB701690)
Australia-Marijiniup8 (NC_023628)
NY-OI12 (LC664202)
NY-OI4 (LC664201)
NY-N15 (LC664199)
NY-CB4 (LC664189)
NY-YM2 (LC664205)
NY-KG6 (LC664206)
NY-KM9 (LC664197)
Group 1
NY-GM252 (LC664193)
NY-SM69A (LC664204)
Group 4
NY-CB3 (LC664188)
NY-KW4 (LC664198)
China-Zhangzhou (JQ326210)
NY-AC230 (LC664186)
NY-CB1 (LC664187)
Group 5
NY-A65 (LC664185)
NY-M4 (LC664184)
NY-F8 (LC664191)
NY-NI263 (LC664200)
NY-HO42 (LC664195)
Group 3
NY-FI29 (LC664182)
NY-FI30 (LC664183)
Australia-Marijiniup9 (JX156421)
NY-HR39 (LC664196)
NY-FK266 (LC664192)
NY-OS1 (LC664203)
NY-HG25 (LC664194)
NY-F1 (LC664190)
Group 2
0.1
C. nt 7700-9651 (3' end)
NYSV (JQ395042)
NYSV (NC_011541)
NYSV (JQ911732)
ScaMV (NC_003399)
TuMV (AB701690)
JYMV (KJ701427)
NY-OI12 (LC664202)
NY-KG6 (LC664206)
NY-N15 (LC664199)
NY-OI4 (LC664201)
NY-CB4 (LC664189)
NY-YM2 (LC664205)
NY-KM9 (LC664197)
Australia-Marijiniup8 (NC_023628)
Group 1
NY-HO42 (LC664195)
NY-NI263 (LC664200)
NY-A65 (LC664185)
NY-F8 (LC664191)
NY-M4 (LC664184)
Group 3
NY-GM252 (LC664193)
NY-SM69A (LC664204)
Group 4
NY-CB3 (LC664188)
NY-KW4 (LC664198)
NY-AC230 (LC664186)
China-Zhangzhou (JQ326210)
Group 5
NY-FI29 (LC664182)
NY-FI30 (LC664183)
NY-HG25 (LC664194)
NY-HR39 (LC664196)
NY-F1 (LC664190)
NY-FK266 (LC664192)
NY-OS1 (LC664203)
Group 2
0.1
D. nt 8617-9438 (coat protein)
NYSV (JQ911732)
NYSV (NC_011541)
NYSV (JQ395042)
ScaMV (NC_003399)
TuMV (AB701690)
JYMV (KJ701427)
NY-CB3 (LC664188)
NY-KW4 (LC664198)
NY-AC230 (LC664186)
China-Zhangzhou (JQ326210)
Clade 4, Group 5
NY-GM252 (LC664193)
NY-SM69A (LC664204)
Clade 4, Group 4
NY-M4 (LC664184)
NY-A65 (LC664185)
NY-F8 (LC664191)
NY-HO42 (LC664195)
NY-NI263 (LC664200)
Clade 3, Group 3
NY-OI12 (LC664202)
NY-N15 (LC664199)
NY-OI4 (LC664201)
NY-KG6 (LC664206)
NY-YM2 (LC664205)
NY-CB4 (LC664189)
Australia-Marijiniup8 (NC_023628)
NY-KM9 (LC664197)
Clade 1, Group 1
NY-FI29 (LC664182)
NY-FI30 (LC664183)
Australia-Marijiniup9 (JX156421)
NY-HR39 (LC664196)
NY-OS1 (LC664203)
NY-F1 (LC664190)
NY-FK266 (LC664192)
NY-HG25 (LC664194)
Clade 2, Group 2
0.1

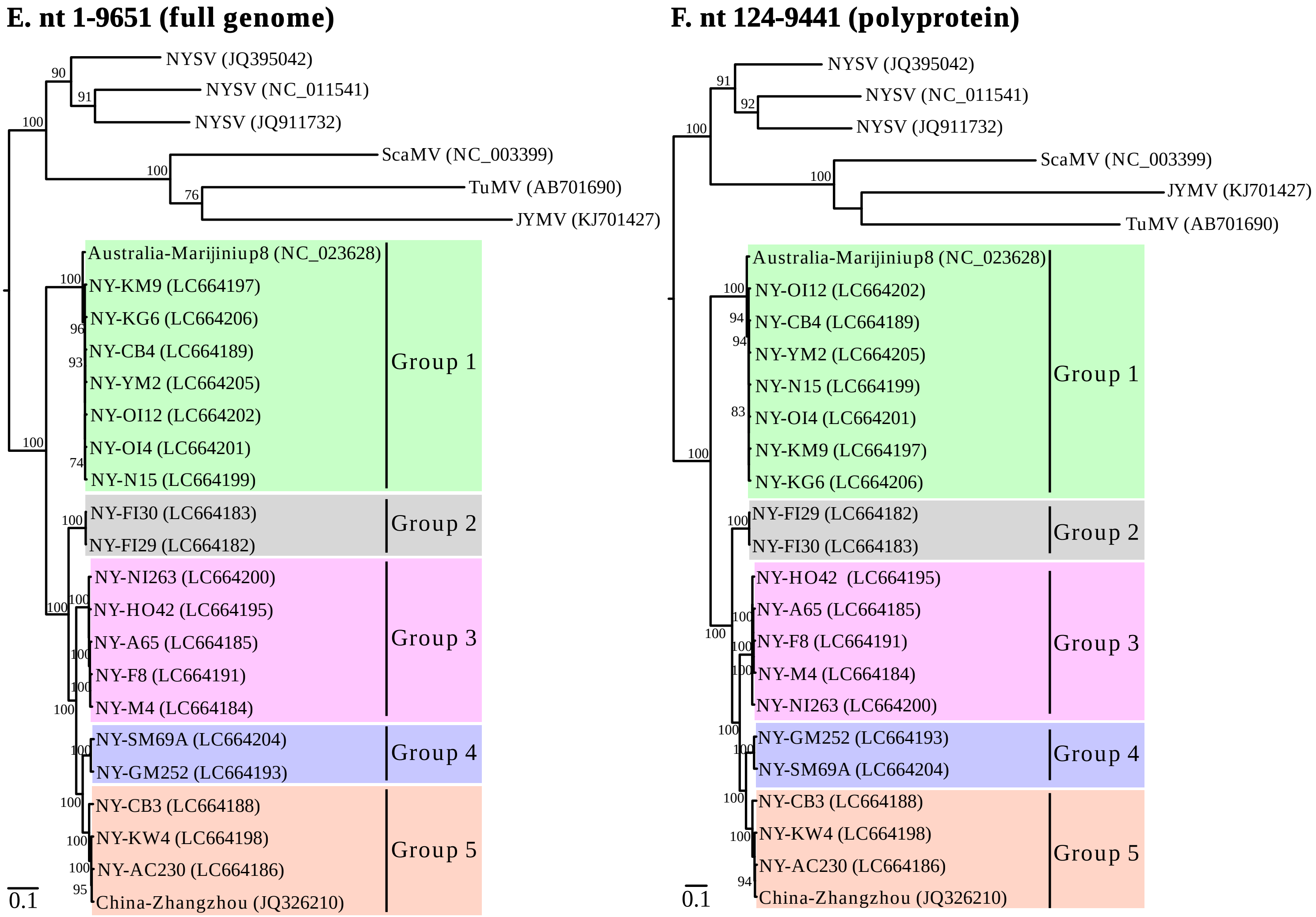

Figure S2. Maximum likelihood phylogenetic trees inferred using full and partial genomic sequences of narcissus late season yellows virus (NLSYV). A. nt 1-1800 region, B. nt 2200-7400 region, C. nt 7700-9651 region (excluding NY-CB1 and Marijiniup9 recombinants), D. nt 8617-9438 region (coat protein coding region, excluding NY-CB1 recombinant), E. nt 1-9651 region (full genomic region, excluding Australia-Marijiniup9, NY-F1, NY-FK226, NY-HG25, NY-HR39, NY-OS1 and NY-CB1 recombinants), and F. nt 124-9441 region (polyprotein coding region, excluding Australia-Marijiniup9, NY-F1, NY-FK226, NY-HG25, NY-HR39, NY-OS1, and NY-CB1 recombinants). The corresponding sequences of Japanese yam mosaic virus (JYMV) [46], narcissus yellow stripe virus (NYSV) [23,26,27], scallion mosaic virus (ScaMV) [48], and turnip mosaic virus (TuMV) [47] in the TuMV phylogenetic group were used as outgroup taxa. Numbers at each node indicate bootstrap percentages based on 1000 pseudoreplicates for all trees and the values >70% are only shown.

Table S1. Collection sites and the results of genetic diagnosis of narcissus plants in this study.

Plant (Isolate)[1]	Location (town, city, prefecture)	Collection date	Host	Symptom	Number of clones partially sequenced[2] (number of clones fully sequenced[3])						
					Total	NDV	NLSYV[2]	NYSV	CyEVA	OrMV	NLV
	**Hokkaido district (island)**										
NY-HO1	Soen, Chuo-ku, Sapporo	20 June 2012	*Narcissus tazzeta* var. *chinensis*	Stripe	7		7 (3)				
NY-HO2	Soen, Chuo-ku, Sapporo	20 June 2012	*N. tazzeta* var. *chinensis*	Stripe	ND[4]						
NY-HO3	Tonden, Kita-ku, Sapporo	21 June 2012	*N. tazzeta* var. *chinensis*	Stripe	ND						
NY-HO4	Tonden, Kita-ku, Sapporo	21 June 2012	*N. tazzeta* var. *chinensis*	Stripe	ND						
NY-HO5	Tonden, Kita-ku, Sapporo	24 June 2012	*N. tazzeta* var. *chinensis*	Stripe	ND						
NY-HO42	Nango, Shiroishi-ku, Sapporo	1 June 2013	*N. tazzeta* var. *chinensis*	Stripe	9		9 (3)				
NY-HO43	Nango, Shiroishi-ku, Sapporo	1 June 2013	*N. tazzeta* var. *chinensis*	Mild stripe	8		8 (3)				
NY-HO44	Kita 48 jo, Kita-ku, Sapporo	2 June 2013	*N. tazzeta* var. *chinensis*	Mild stripe	8		8 (3)				
NY-HO45	Fujino, Minami-ku, Sapporo	2 June 2013	*N. tazzeta* var. *chinensis*	Mild stripe	ND						
NY-HO46	Fujino, Minami-ku, Sapporo	2 June 2013	*N. tazzeta* var. *chinensis*	Mild stripe	ND						
NY-HO47	Kitanosawa, Minami-ku, Sapporo	2 June 2013	*N. tazzeta* var. *chinensis*	Mild stripe	ND						
NY-HO48	Maeda, Teine-ku, Sapporo	2 June 2013	*N. tazzeta* var. *chinensis*	Symptomless	8				8		
NY-HO49	Tonden, Kita-ku, Sapporo	3 June 2013	*Narcissus bulbocodium*	Symptomless	ND						
NY-HO50	Tonden, Kita-ku, Sapporo	3 June 2013	*N. tazzeta* var. *chinensis*	Mild stripe	ND						
NY-HO51	Tonden, Kita-ku, Sapporo	3 June 2013	*N. tazzeta* var. *chinensis*	Mild stripe	ND						
NY-HO52	Tonden, Kita-ku, Sapporo	3 June 2013	*N. tazzeta* var. *chinensis*	Mild stripe	10		10 (3)				
NY-HO54	Minaminosato, Kitahirosima	3 June 2013	*N. tazzeta* var. *chinensis*	Mild stripe	ND						
NY-HO55	Higashikyouei, Kitahirosima	3 June 2013	*N. tazzeta* var. *chinensis*	Mild stripe	ND						
NY-HO56	Shinano, Chitose	3 June 2013	*N. tazzeta* var. *chinensis*	Mild stripe	ND						
NY-HO186	Shinonome-cho, Chitose	4 June 2014	*Narcissus tazzeta?*	Severe stripe	9						9
NY-HO301	Maruyama, Kuriyama-cho, Yubari-gun,	6 May 2015	*Narcissus jonquilla*	Severe mosaic, stripe	9						9
NY-HO306	Honcho, Naname-cho, Kameda-gun	6 May 2015	*Narcissus pseudonarcisuus*	Symptomless	ND						
NY-HO307	Funamicho, Toyoura-cho, Abuta-gun	7 May 2015	*N. pseudonarcisuus*	Mild stripe	10		10 (3)				
NY-HO308	Fukushima, KImobetsu-cho, Abuta-gun	7 May 2015	*N. tazzeta* var. *chinensis*	Symptomless	ND						
	**Tohoku district**										
NY-A65	Nanbu-cho, Sannohe-gun, Aomori	22 January 2014	*N. tazzeta* var. *chinensis*	Mosaic	10		10 (3)				
NY-A178	Nanbu-cho, Sannohe-gun, Aomori	15 May 2014	*N. tazzeta* var. *chinensis*	Mosaic	11		11 (3)				
NY-IW1	-, Morioka, Iwate	14 June 2014	*N. tazzeta* var. *chinensis*	Symptomless	ND						
NY-IW2	-, Morioka, Iwate	14 June 2014	*N. tazzeta* var. *chinensis*	Symptomless	ND						
NY-IW148	Toyama, Shiwa-cho, Shiwa-gun, Iwate	14 June 2014	*N. jonquilla*		10			10			
NY-IW149	Toyama, Shiwa-cho, Shiwa-gun, Iwate	14 June 2014	*N. jonquilla*	Stripe	10			10			
NY-IW196	Koromogawa-ku, Oshu, Iwate	14 June 2014	*N. tazzeta* var. *chinensis*	Stripe	ND						
NY-IW203	Kawashiri, Nishiwaga-machi, Waga-gun, Iwate	14 June 2014	*N. tazzeta* var. *chinensis*	Severe stripe	ND						
NY-IW204	Kamiezuriko, Kitakami, Iwate	14 June 2014	*N. tazzeta* var. *chinensis*	Stripe	ND						
NY-MY210	Irimada, Shibata-machi, Shibata-gun, Miyagi	14 June 2014	*N. tazzeta* var. *chinensis*	Stripe	ND						
NY-MY211	Minamihase, Iwanuma, Miyagi	14 June 2014	*N. tazzeta* var. *chinensis*	Symptomless	ND						
NY-MY292	Zao-machi, Katta, Miyagi	23 April 2015	*N. tazzeta* var. *chinensis*	Symptomless	8		8 (2)				
NY-MY293	Zao-machi, Katta, Miyagi	23 April 2015	*N. tazzeta* var. *chinensis*	Mosaic, stripe	10		3 (2)	5			2
NY-AK205	Sugisawaaratokoro, Yuzawa, Akita	14 June 2014	*N. tazzeta* var. *chinensis*	Stripe	ND						
NY-AK285	Hiroomote, Hiroomote, Akita Akita	25 April 2015	*N. tazzeta* var. *chinensis*	Stripe	ND						
NY-AK287	Nishinagano, Kadonodate-machi, Senboku, Akita	25 April 2015	*N. tazzeta* var. *chinensis*	Mild stripe	ND						
NY-AK288	Sotsuda, Kitazawako, Senboku, Akita	25 April 2015	*N. jonquilla*	Mild stripe	ND						
NY-AK289	Kyowahunooka, Daisen, Akita	25 April 2015	*N. jonquilla*	Mild stripe	ND						
NY-YA193	Zaohotta, Yamagata, Yamagata	15 June 2014	*N. tazzeta* var. *chinensis*	Symptomless	ND						
NY-YA194	Hara-machi, Tendo, Yamagata	15 June 2014	*N. tazzeta* var. *chinensis*	Stripe	ND						
NY-YA195	Kubota-machi, Yonezawa, Yamagata	15 June 2014	*N. tazzeta* var. *chinensis*	Stripe	ND						
NY-YA276	-, Tsuruoka, Yamagata	25 April 2015	*N. tazzeta* var. *chinensis*	Mild stripe	ND						

[1] Rows in brown show that narcissus plants were co-infected with two or more viruses, whereas rows in blue show that those were singley infected with narcissus degeneration virus (NDV), narcissus late season yellows virus (NLSYV), narcissus yellow stripe virus (NYSV)-like virus, cyrtanthus elatus virus A (CyEVA), ornithogalum mosaic virus (OrMV) or narcissus latent virus (NLV).

[2] Number of clones sequenced for approximately 650-700 bp by POTYNIB5P primer.

[3] Number of clones sequenced for coat protein coding region.

[4] Not detected.

Table S1. Continued.

Plant (Isolate)	Location (town, city, prefecture)	Collection date	Host	Symptom	Number of clones partially sequenced[2] (number of clones fully sequenced[3])						
					Total	NDV	NLSYV	NYSV	CyEVA	OrMV	NLV
NY-FK266	Motomiya, Fukushima	23 April 2015	*N. jonquilla*	Stripe	18	12	2 (2)	4			
NY-FK270	Akogashima, Atami-machi, Koriyama, Fukushima	23 April 2015	*N. tazzeta* var. *chinensis*	Symptomless	ND						
NY-FK275	Yamagata, Inawashiro-machi, Fukushima, Fukushima	23 April 2015	*N. jonquilla*	Mild stripe	ND						
	**Kanto district**										
NY-IB71A	-, -, Ibaraki	11 November 2013	*Narcissus* sp. cv. Erlicheer	Stripe	10		10 (2)				
NY-IB248	Kanaya-cho, Mito, Ibaraki	17 March 2015	*N. tazzeta* var. *chinensis*	Stripe	9				9		
NY-TC249	Ota, Fujioka-machi, Tochigi, Tochigi	17 March 2015	*N. tazzeta* var. *chinensis*	Yellowing	ND						
NY-TC250	Osawa, Mashiko-machi, Haga-gun, Tochigi	17 March 2015	*N. jonquilla*	Symptomless	10				10		
NY-TC251	Nogoya-machi, Utsunomiya, Tochigi	17 March 2015	*N. tazzeta* var. *chinensis*	Yellowing	10	1 (1)	9 (4)				
NY-GM252	Fujiki, Tomioka, Gunma	17 March 2015	*N. tazzeta* var. *chinensis*	Mild stripe	8		8 (3)				
NY-GM253	Fujiki, Tomioka, Gunma	17 March 2015	*N. tazzeta* var. *chinensis*	Mild stripe	ND						
NY-GM254	Shiroishi, Fujioka, Gunma	17 March 2015	*N. tazzeta* var. *chinensis*	Severe stripe	ND						
NY-ST1	Kamifukuoka, Fujimino, Saitama	28 March 2010	*N. tazzeta* var. *chinensis*	Mild stripe	9		8 (0)	1			
NY-ST2	-, Toda, Saitama	30 March 2010	*N. tazzeta* var. *chinensis*	Mild stripe	9	2 (2)	5 (0)	2			
NY-CB1	Tokashiro, Kimitsu, Chiba	26 March 2010	*N. tazzeta* var. *chinensis*	Mild stripe	8		7 (7)	1			
NY-CB2	Sotominowa, Kimitsu, Chiba	26 March 2010	*N. tazzeta* var. *chinensis*	Mild stripe	10		2 (1)	8			
NY-CB3	Sotominowa, Kimitsu, Chiba	26 March 2010	*N. tazzeta* var. *chinensis*	Mild stripe	10		9 (9)	1			
NY-CB4	Ezuki, Kyonan-machi, Awa-gun, Chiba	26 March 2010	*N. tazzeta* var. *chinensis*	Mild stripe	10		10 (9)				
NY-CB5	Ezuki, Kyonan-machi, Awa-gun, Chiba	26 March 2010	*N. tazzeta* var. *chinensis*	Stripe	10	2 (2)	7 (6)	1			
NY-CB9	Ezuki, Kyonan-machi, Awa-gun, Chiba	26 March 2010	*N. tazzeta* var. *chinensis*	Mild stripe	9		4 (4)	5			
NY-CB91	Ezuki, Kyonan-machi, Awa-gun, Chiba	22 November 2013	*N. tazzeta* var. *chinensis*	Severe stripe	18	13 (5)	5 (2)				
NY-CB247	Ezuki, Kyonan-machi, Awa-gun, Chiba	17 March 2015	*N. tazzeta* var. *chinensis*	Stripe	10		4 (2)	6			
NY-TK2	-, Koutou-ku, Tokyo	25 February 2010	*N. tazzeta* var. *chinensis*	Mild stripe	10		10 (10)				
NY-TK4	-, Akishima, Tokyo	28 March 2010	*N. tazzeta* var. *chinensis*	Mild stripe	10		5 (4)	5			
NY-TK243	-, Ota-ku, Tokyo	17 March 2015	*N. tazzeta* var. *chinensis*	Severe stripe	10		10 (3)				
NY-KN1	Hasse-machi, Miura, Kanagawa	27 March 2010	*N. tazzeta* var. *chinensis*	Mild stripe	18		10 (3)			8	
NY-KN2	Jogashima, Misaki, Miura, Kanagawa	27 March 2010	*N. tazzeta* var. *chinensis*	Symptomless	ND						
NY-KN3	Jogashima, Misaki, Miura, Kanagawa	27 March 2010	*N. tazzeta* var. *chinensis*	Symptomless	ND						
NY-KN4	Jogashima, Misaki, Miura, Kanagawa	27 March 2010	*N. tazzeta* var. *chinensis*	Symptomless	ND						
NY-KN5	Jogashima, Misaki, Miura, Kanagawa	27 March 2010	*N. tazzeta* var. *chinensis*	Symptomless	ND						
NY-KN6	Oba, Fujisawa, Kanagawa	27 March 2010	*N. tazzeta* var. *chinensis*	Mild stripe	9		8 (8)	1			
NY-KN7	Oba, Fujisawa, Kanagawa	27 March 2010	*N. tazzeta* var. *chinensis*	Mild stripe	9		9 (3)				
NY-KN8	Sekiguchi, Atsugi, Kanagawa	28 March 2010	*N. tazzeta* var. *chinensis*	Mild stripe	8		8 (3)				
NY-KN9	-, Atsugi, Kanagawa	28 March 2010	*N. tazzeta* var. *chinensis*	Mild stripe	9		9 (3)				
	**Chubu district**										
NY-NI7	-, -, Niigata	5 April 2010	*Narcissus tazzeta* var. Scarlet gem	Symptomless	ND						
NY-NI260	Osato, Agano, Niigata	23 April 2015	*N. tazzeta* var. *chinensis*	Mild stripe	ND						
NY-NI263	Kuraoka, Konan-ku, Niigata, Niigata	24 April 2015	*N. tazzeta* var. *chinensis*	Mild stripe	9		9 (2)				
NY-TY73A	-, -, Toyama	11 November 2013	*N. tazzeta*	Symptomless	4		4 (3)				
NY-FI3	Hamakitayama, Fukui, Fukui	17 January 2012	*N. tazzeta* var. *chinensis*	Mild stripe	8	2 (2)	7 (5)				
NY-FI9	Hamakitayama, Fukui, Fukui	17 January 2012	*N. tazzeta* var. *chinensis*	Mild stripe	17	13 (6)	1 (1)	3			
NY-FI11	Hamakitayama, Fukui, Fukui	17 January 2012	*N. tazzeta* var. *chinensis*	Mild stripe	10	3 (3)	7 (6)				
NY-FI15	Gamo, Fukui, Fukui	8 March 2012	*N. tazzeta* var. *chinensis*	Mild stripe	ND						
NY-FI23	Hamakitayama, Fukui, Fukui	8 March 2012	*N. tazzeta* var. *chinensis*	Stripe	9	9 (5)					
NY-FI29	Nashigadaira, Echizen-cho, Nyuu, Fukui	8 March 2012	*N. tazzeta* var. *chinensis*	Stripe	10		10 (7)				
NY-FI30	Nashigadaira, Echizen-cho, Nyuu, Fukui	8 March 2012	*N. tazzeta* var. *chinensis*	Stripe	9	1 (1)	8 (8)				
NY-FI33	Chigadaira, Echizen-cho, Nyuu, Fukui	8 March 2012	*N. tazzeta* var. *chinensis*	Stripe	18	8 (2)	10 (2)				
NY-NN75A	Hananoyamato, Daiichi Engei, Nagano	11 November 2013	*Narcissus tazzeta odoru*		ND						

Table S1. Continued.

Plant (Isolate)	Location (town, city, prefecture)	Collection date	Host	Symptom	Number of clones partially sequenced[2] (number of clones fully sequenced[3])						
					Total	NDV	NLSYV	NYSV	CyEVA	OrMV	NLV
NY-SH216	Suzaki, Shimoda, Shizuoka	7 December 2014	*N. tazzeta* var. *chinensis*	Mosaic	10			10			
NY-SH219	Komakado, Gotemba, Shizuoka	7 December 2014	*N. tazzeta* var. *chinensis*	Stripe	10		10 (2)				
NY-SH236	Takasaki, Yaizu, Shizuoka	25 January 2015	*N. tazzeta* var. *chinensis*	Severe stripe	10		9 (2)				
NY-AC230	Sannomaru, Naka-ku, Nagoya, Aichi	25 January 2015	*N. tazzeta* var. *chinensis*	Severe stripe	19	13 (3)	2 (2)	4			
NY-AC233	Zoshi, Toyokawa, Aichi	25 January 2015	*N. tazzeta* var. *chinensis*	Severe stripe	9	2 (2)	7 (2)				
NY-AC234	Nagasawa-cho, Toyokawa, Aichi	25 January 2015	*N. tazzeta* var. *chinensis*	Mild stripe	ND						
	**Kinki district**										
NY-ME224	Koazaka-cho, Matsuzaka, Mie	24 January 2015	*N. tazzeta* var. *chinensis*	Stripe	9		9 (2)				
NY-ME226	Shihikida, Taki-cho, Taki-gun, Mie	24 January 2015	*N. tazzeta* var. *chinensis*	Stripe	ND						
NY-ME229	Komei-cho, Tsu, Mie	24 January 2015	*N. tazzeta* var. *chinensis*	Stripe	19	17 (7)		2			
NY-KY83	-, -, Kyoto	19 November 2013	*Narcissus bulbocidum*	Symptomless	ND						
NY-KY84	-, -, Kyoto	19 November 2013	*N. bulbocidum*	Symptomless	ND						
NY-OS1	Minowa, Toyonaka, Osaka	10 March 2012	*N. tazzeta* var. *chinensis*	Stripe	7		4 (3)	3			
NY-OS2	Himemuro, Ikeda, Osaka	10 March 2012	*N. tazzeta* var. *chinensis*	Stripe	7		7 (3)				
NY-HG1	-, Awaji, Hyogo	12 March 2011	*N. tazzeta* var. *chinensis*	Mild stripe	ND						
NY-HG3	Ichinomiya, Awaji, Hyogo	12 March 2011	*N. pseudonarcissus*	Stripe	8			4	4		
NY-HG5	Ichinomiya, Awaji, Hyogo	12 March 2011	*N. tazzeta* var. *chinensis*	Severe mosaic	ND						
NY-HG6	Ichinomiya, Awaji, Hyogo	12 March 2011	*N. tazzeta* var. *chinensis*	Severe mosaic	ND						
NY-HG8	Nadakuroiwa, Minamiawaji, Hyogo	12 March 2011	*N. tazzeta* var. *chinensis*	Stripe	16	9 (6)	1 (1)	6			
NY-HG10	Nadakuroiwa, Minamiawaji, Hyogo	12 March 2011	*N. tazzeta* var. *chinensis*	Stripe	10	7 (4)	2 (1)	1			
NY-HG11	Yura-cho, Sumoto, Hyogo	12 March 2011	*N. tazzeta* var. *chinensis*	Stripe	10		10 (0)				
NY-HG13	Hon-machi, Himeji, Hyogo	14 March 2011	*N. tazzeta* var. *chinensis*	Mosaic, stripe	ND						
NY-HG16	Amida-cho, Takasago, Hyogo	14 March 2011	*N. tazzeta* var. *chinensis*	Stripe	6		1 (3)	5			
NY-HG19	Kukuchinishi-cho, Amagasaki, Hyogo	10 March 2012	*N. tazzeta* var. *chinensis*	Stripe	11			11			
NY-HG20	Meishin-cho, Amagasaki, Hyogo	10 March 2012	*N. tazzeta* var. *chinensis*	Stripe	9	8 (7)	1 (1)				
NY-HG25	Akurakita, Takarazuka, Hyogo	10 March 2012	*N. tazzeta* var. *chinensis*	Stripe	10		10 (8)				
NY-HG24	Sakae, Takarazuka, Hyogo	10 March 2012	*N. tazzeta* var. *chinensis*	Stripe	ND						
NY-HG27	Kamo, Kawanishi, Hyogo	10 March 2012	*N. tazzeta* var. *chinensis*	Stripe	16			16			
	**Chugoku district**										
NY-SM69A	Unknown, Shimane	11 November 2013	*Narcissus bulbocidum* var. *monoohyllus*	Symptomless	10		1 (1)			9	
NY-OY1	Kotsu, Higashi-ku, Okayama, Okayama	13 March 2011	*N. tazzeta* var. *chinensis*	Stripe	9		4 (1)	5			
NY-OY41	Kuma, Kita-ku, Okayama, Okayama	9 March 2013	*N. tazzeta* var. *chinensis*	Mild stripe	ND						
NY-HR36	Yanohigashi, Akiku, Hiroshima, Hiroshima	8 March 2013	*N. tazzeta* var. *chinensis*	Mild stripe	7			7			
NY-HR38	Ushirogake, Kawajiri, Kure, Hiroshima	8 March 2013	*N. tazzeta* var. *chinensis*	Mild stripe	9			9			
NY-HR39	Sunami, Mihara, Hiroshima	8 March 2013	*N. tazzeta* var. *chinensis*	Mild stripe	7		7 (3)				
NY-HR40	Higashiteshiro, Fukuyama, Hiroshima	9 March 2013	*N. tazzeta* var. *chinensis*	Mild stripe	20	20 (6)					
NY-YM2	Chinto, Hagi, Yamaguchi	26 February 2011	*N. tazzeta* var. *chinensis*	Stripe	10	6 (5)	4 (4)				
NY-YM5	Nabe-cho, Shimonoseki, Yamaguchi	27 February 2011	*N. tazzeta* var. *chinensis*	Stripe	10	5 (5)	3 (2)	2			
	**Shikoku district**										
NY-TS158	Kizuno, Otsu-cho, Naruto, Tokushima	24 April 2014	*N. tazzeta* var. *chinensis*	Symptomless	3						3
NY-TS159	Kizuno, Otsu-cho, Naruto, Tokushima	24 April 2014	*N. tazzeta* var. *chinensis*	Symptomless	ND						
NY-TS160	Yoshinaga, Otsu-cho, Naruto, Tokushima	26 April 2014	*N. tazzeta* var. *chinensis*	Mild stripe	ND						
NY-TS161	-, Mima, Tokushima	26 April 2014	*N. tazzeta* var. *chinensis*	Symptomless	ND						
NY-KW1	Ejiri-cho, Sakaide, Kagawa	13 March 2011	*N. tazzeta* var. *chinensis*	Stripe	13	9 (5)	4 (2)				
NY-KW2	Oumi, Sakaide, Kagawa	13 March 2011	*N. tazzeta* var. *chinensis*	Mild stripe	3				3		
NY-KW3	Oumi, Sakaide, Kagawa	13 March 2011	*N. tazzeta* var. *chinensis*	Stripe	10			10			
NY-KW4	Ikushima-cho, Takamatsu, Kagawa	13 March 2011	*N. tazzeta* var. *chinensis*	Stripe	8		8 (3)				
NY-KW5	Nakayama-cho, Takamatsu, Kagawa	13 March 2011	*N. pseudonarcissus*	Mosaic	7					7	
NY-KW6	Danshi-cho, Takamatsu, Kagawa	13 March 2011	*N. tazzeta* var. *chinensis*	Mosaic	8			8			
NY-EH171	Takanoko-cho, Matsuyama, Ehime	26 April 2014	*N. tazzeta* var. *chinensis*	Stripe	10	3 (2)		7			
NY-EH173	Kurumi, Tambara-cho, Saijo, Ehime	26 April 2014	*N. tazzeta* var. *chinensis*	Severe stripe	10		10 (0)				
NY-KO163	Otesuji, Kochi, Kochi	25 April 2014	*N. tazzeta* var. *chinensis*	Unclear	ND						
NY-KO164	Aioi-cho, Kochi, Kochi	25 April 2014	*N. tazzeta* var. *chinensis*	Unclear	9			9			
NY-KO165	Ikku, Kochi, Kochi	25 April 2014	*N. tazzeta* var. *chinensis*	Stripe	ND						

Table S1. Continued.

Plant (Isolate)	Location (town, city, prefecture)	Collection date	Host	Symptom	Number of clones partially sequenced[2] (number of clones fully sequenced[3])						
					Total	NDV	NLSYV	NYSV	CyEVA	OrMV	NLV
	**Kyushu and Okinawa district (islands)**										
NY-F1	-, Okawa, Fukuoka	20 March 2010	*N. tazzeta* var. *chinensis*	Mosaic	10	4 (3)	3 (3)	3			
NY-F2	Yamakawa-machi, Miyama, Fukuoka	24 January 2011	*N. tazzeta* var. *chinensis*	Mild stripe	7		7 (3)				
NY-F8	Kawabaru, Itoshima, Fukuoka	11 December 2011	*N. tazzeta* var. *chinensis*	Mild yellow stripe	9		9 (3)				
NY-F9	Misaka, Itoshima, Fukuoka	11 December 2011	*N. tazzeta* var. *chinensis*	Mild yellow stripe	6			6			
NY-SG8	Hyogo-machi, Saga, Saga	14 March 2010	*N. tazzeta* var. *chinensis*	Mosaic	10	8 (8)	2 (2)				
NY-SG10	Honjo-machi, Saga, Saga	14 January 2011	*N. tazzeta* var. *chinensis*	Mosaic, stripe	ND						
NY-SG12	Honjo-machi, Saga, Saga	8 January 2013	*N. tazzeta* var. *chinensis*	Stripe	9	6 (3)	3 (3)				
NY-N2	Kunimi-cho, Unzen, Nagasaki	6 March 2009	*N. tazzeta* var. *chinensis*	Symptomless	ND						
NY-N6	Kayaki-cho, Nagasaki, Nagasaki	27 February 2010	*N. tazzeta* var. *chinensis*	Mild yellow stripe	ND						
NY-N9	Kurohama-machi, Nagasaki, Nagasaki	27 February 2010	*N. tazzeta* var. *chinensis*	Mosaic	11	9 (9)	2 (2)				
NY-N12	Nomozaki, Nomo-cho, Nagasaki, Nagasaki	27 February 2010	*N. tazzeta* var. *chinensis*	Yellow stripe	8		8 (3)				
NY-N13	Nomozaki, Nomo-cho, Nagasaki, Nagasaki	27 February 2010	*N. tazzeta* var. *chinensis*	Mild yellow stripe	ND						
NY-N14	Nomozaki, Nomo-cho, Nagasaki, Nagasaki	27 February 2010	*N. tazzeta* var. *chinensis*	Mosaic	15	6 (2)	1 (1)	7			
NY-N15	Uki-machi, Isahaya, Nagasaki	27 February 2010	*N. tazzeta* var. *chinensis*	Yellow stripe	10		4 (3)	6			
NY-N18	Obama-cho, Unzen, Nagasaki	28 February 2010	*N. tazzeta* var. *chinensis*	Yellow stripe	ND						
NY-N19	Arima-cho, Minamishimabara, Nagasaki	28 February 2010	*N. tazzeta* var. *chinensis*	Yellow stripe	15		4 (3)	1	10		
NY-N20	Ariake-cho, Shimabara, Nagasaki	28 February 2010	*N. tazzeta* var. *chinensis*	Yellow stripe	10			10			
NY-KM1O	Miyabara, Arao, Kumamoto	23 January 2011	*N. tazzeta* var. *chinensis*	Mild stripe	10			10			
NY-KM1P	Miyabara, Arao, Kumamoto	23 January 2011	*N. tazzeta* var. *chinensis*	Mild stripe	10			10			
NY-KM9	Ozu-machi, Kikuchi-gun, Kumamoto	28 March 2011	*N. tazzeta* var. *chinensis*	Severe stripe	10	8 (7)	2 (2)				
NY-OI1	Takasakiyama, Kozaki, Oita, Oita	26 February 2012	*N. tazzeta* var. *chinensis*	Yellow stripe	10			10			
NY-OI2	Takasakiyama, Kozaki, Oita, Oita	26 February 2012	*N. tazzeta* var. *chinensis*	Yellow stripe	10			10			
NY-OI3	Takasakiyama, Kozaki, Oita, Oita	26 February 2012	*N. tazzeta* var. *chinensis*	Yellow stripe	10			10			
NY-OI4	Tanoyu-machi, Beppu, Oita	26 February 2012	*N. tazzeta* var. *chinensis*	Yellow stripe	11		3 (3)	8			
NY-OI5	Tanoyu-machi, Beppu, Oita	26 February 2012	*N. tazzeta* var. *chinensis*	Yellow stripe	8			8			
NY-OI12	Fujimi-cho, Beppu, Oita	26 February 2012	*N. tazzeta* var. *chinensis*	Yellow stripe	9		3	6 (3)			
NY-OI13	Tenman-cho, Beppu, Oita	26 February 2012	*N. tazzeta* var. *chinensis*	Yellow stripe	10			10			
NY-OI14	Tenman-cho, Beppu, Oita	26 February 2012	*N. tazzeta* var. *chinensis*	Mild yellow stripe	ND						
NY-M1	Higashinagaeura, Ebino, Miyazaki	7 May 2012	*N. tazzeta* var. *chinensis*	Mosaic	3						3
NY-M2	Higashinagaeura, Ebino, Miyazaki	7 May 2012	*N. tazzeta* var. *chinensis*	Yellow stripe	7			6	1		
NY-M3	Suenaga, Ebino, Miyazaki	7 May 2012	*N. tazzeta* var. *chinensis*	Yellow stripe	ND						
NY-M4	Takaharu, Nishimorokata-gun, Miyazaki	7 May 2012	*N. tazzeta* var. *chinensis*	Severe yellow stripe	10		10 (5)				
NY-M5	Takazaki-cho, Miyakonojo, Miyazaki	7 May 2012	*N. tazzeta* var. *chinensis*	Yellow stripe	ND						
NY-M77	-, -, Miyazaki	15 November 2013	*N. tazzeta* var. *chinensis*	Stripe	10		10 (4)				
NY-KG1	Tokunoshima-cho, Oshima-gun, Kagoshima	22 January 2010	*N. tazzeta* var. *chinensis*	Yellow stripe	10			10			
NY-KG2	Tokunoshima-cho, Oshima-gun, Kagoshima	22 January 2010	*N. tazzeta* var. *chinensis*	Mild yellow stripe	9		6 (3)	3			
NY-KG6	Tenokuchi, Kagoshima, Kagoshima	16 February 2012	*N. tazzeta* var. *chinensis*	Yellow stripe	9		9 (3)				
NY-KG7	Tenokuchi, Kagoshima, Kagoshima	16 February 2012	*N. tazzeta* var. *chinensis*	Yellow stripe	ND						
NY-KG8	Yamanokuchi, Kagoshima, Kagoshima	16 February 2012	*N. tazzeta* var. *chinensis*	Yellow stripe	ND						
NY-KG9	Uenosono-cho, Kagoshima, Kagoshima	16 February 2012	*N. tazzeta* var. *chinensis*	Yellow stripe	6		6 (3)				
NY-KG10	Uearata-cho, Kagoshima, Kagoshima	16 February 2012	*N. tazzeta* var. *chinensis*	Severe yellow stripe	13	12 (6)		1			
NY-KG11	Uearata-cho, Kagoshima, Kagoshima	16 February 2012	*N. tazzeta* var. *chinensis*	Yellow stripe	6	6 (2)					
NY-KG12	Arata-cho, Kagoshima, Kagoshima	16 February 2012	*N. tazzeta* var. *chinensis*	Yellow stripe	9			9			
NY-KG13	Uearata-cho, Kagoshima, Kagoshima	16 February 2012	*N. tazzeta* var. *chinensis*	Yellow stripe	10		10 (3)				
NY-OK98	Kyoda, Nago, Okinawa	25 January 2014	*N. tazzeta* var. *chinensis*	Severe mosaic	10			10			
NY-OK100	Kyoda, Nago, Okinawa	25 January 2014	*N. tazzeta* var. *chinensis*	Stripe	6			6			

Table S2. Primers used for RT-PCR amplification and sequencing in this study.

A. Narcissus late season yellows virus and potyvirus specific primers used for RT-PCR amplification.

Primer name	Type	Position[1]	Region[2]	Sequence (5'-3')[3]
TU5TNOT5P	Forward	1-24	5'NCR	GGGGCGGCCGCAAAAAATATAAAAACTCAACACAACA
TU5TNOT500P	Forward	1-25	5'NCR	GGGGCGGCCGCAAAAATATAAAAACTCAACACAACMA
POTYP1PONOT38P	Forward	3073-3094	P3	GGGGCGGCCGCATATGGGGTGAGAGAGGATTTARRMGGCAGATACRGCG
NLCINOT272M	Reverse	3819-3840	CI	GGGGCGGCCGCTTCTGTTCGATAATGCGGCACC
NLCINOT204M	Reverse	3842-3863	CI	GGGGCGGCCGCCTYGTRAATGTYACSAGATGTC
NLCINOT205M	Reverse	3842-3863	CI	GGGGCGGCCGCCTCGTGAATGTCACGAGATGTC
NLCINOT273M	Reverse	3941-3962	CI	GGGGCGGCCGCGGTAAACCAGTCGATTTTCCAG
NL6K2NOT99P	Forward	5626-5647	6K2	GGGGCGGCCGCTGYCTGGGYATTAAAGGAAGGT
NLVPGNOT100P	Forward	6059-6080	VPg	GGGGCGGCCGCTCACGSTWATRCGAGARGAGAA
NLNIANOT271M	Reverse	6460-6481	NIa-Pro	GGGGCGGCCGCCRAAGAGGTGCCTATTTGTAAG
NLNIANOT129M	Reverse	6586-6607	NIa-Pro	GGGGCGGCCGCCRTCCTTTGGTAAYCTRACAAG
NLNIANOT130M	Reverse	6586-6607	NIa-Pro	GGGGCGGCCGCCATCYTTWGGTAGTCTGACAAG
NLSNIANOT33M	Reverse	6588-6610	NIa-Pro	GGGGCGGCCGCGRACATCYTTWGGTAGTCTGACA
NLNIANOT61P	Forward	6741-6763	NIa-Pro	GGGGCGGCCGCGGGGAGCAATTTTTGGAAACACT
POTYNIBNOT4P	Forward	7670-7691	NIb	GGGGCGGCCGCATATGGGGTGAGAGAGGTNTGYGTNGAYGAYTTYAAYAA
NLNIBNOT146P	Forward	8497-8518	NIb	GGGGCGGCCGCGAGACAGCTTTGCGAAAACTCT
NLCPNOT101M	Reverse	9495-9521	3'NCR	GGGGCGGCCGCHHTYKTGTGATAATATTTCTCTATAGT
NL3TNOT225M	Reverse	9534-9554	3'NCR	GGGGCGGCCGCAAGAGTACGTTCTTGGCTGTG
NL3TNOT196M	Reverse	9534-9554	3'NCR	GGGGCGGCCGCAAGATTACATTCTTGGCAGCG
TU3T9M	Reverse	Poly A	Poly A	GGGGCGGCCGCTTTTTTTTTTTTTTTTTTTTTTTTTTTTTT

[1] Correspond to the position in the genome of Zhangzhou isolate [26].
[2] 5'NCR; 5' non-coding region, P3; third protein, CI; cylindrical inclusion protein, 6K2; 6Kda 2 protein, VPg; genome-linked viral protein, NIa-Pro; nuclear inclusion a protein-proteinase protein, NIb; nuclear inclusion b protein, 3'NCR; 3' non-coding region, Poly A; Poly (A) tail.
[3] R; G+A, Y; C+T, S; G+C, M; A+C, W; A+T, K, T+G, V; A+C+G, D; A+T+G, H; A+T+C, N; A+T+G+C
GCGGCCGC; NotI restriction site

B. Narcissus degeneration virus and potyvirus- and onion yellow dwarf virus phylogenetic group-specific primers used for RT-PCR amplification.

Primer name	Type	Position[1]	Region[2]	Sequence amplification (5'-3')[3]
ND5T4P	Forward	1-23	5'NCR	GGGGCGGCCGCAAATTATAAAATCACATAACAAA
NDHCNOT315P	Forward	1976-1997	HC-Pro	GGGGCGGCCGCCTCTGAATCAACCGATTATGAC
NDHCNOT316M	Reverse	2403-2424	HC-Pro	GGGGCGGCCGCCCAAGGTCTTAACGACTTTGTC
NDHCNOT95P	Forward	2441-2463	HC-Pro	GGGGCGGCCGCGAAAGATGTCGCGACCATGCTAG
NDP3NOT74P	Forward	2810-2832	P3	GGGGCGGCCGCTTGTTTYAAGGAATTGATGGGGG
NDP3NOT317M	Reverse	2820-2841	P3	GGGGCGGCCGCTGAAGATGGCCCCCATCAATTC
NDP3NOT270P	Forward	3165-3186	P3	GGGGCGGCCGCTACGCCGGAGAATCAAACAAGC
NDCINOT269M	Reverse	4014-4035	CI	GGGGCGGCCGCTTATGTCGAAGTCCACAACCAG
NDCINOT97P	Forward	5415-5436	CI	GGGGCGGCCGCTTTGGAGCTCGAGTGCAACTTC
ND6K2NOT288P	Forward	5907-5928	6K2	GGGGCGGCCGCGCTTTGGATCTCAAAGGGACAT
NDVPGNOT96M	Reverse	6486-6508	VPg	GGGGCGGCCGCTGAGCTGGAAACCCAATCTGTCG
NDNIA42M	Reverse	6711-6733	NIa-Pro	GGGGCGGCCGCTGATTGGCAATCATGTATGACCC
RGNDNIBNOT4P	Forward	7724-7744	NIb	GGGGCGGCCGCATATGGGGTGAGAGAGGRTAYSRWGGGAAGAAGAAGGA
POTYNIBNOT4P	Forward	7935-7957	NIb	GGGGCGGCCGCATATGGGGTGAGAGAGGTNTGYGTNGAYGAYTTYAAYAA
NDNIBNOT73M	Reverse	8746-8770	NIb	GGGGCGGCCGCGCTGTATCTGCTAGATAAGGAGCCT
NDCPNOT98M	Reverse	9014-9034	CP	GGGGCGGCCGCGGAAACTTCATCTTGGAACTC
ND3TNOT268M	Reverse	9700-9721	3'NCR	GGGGCGGCCGCACTCCCTAAACTTGTACTTCAC
TU3T9M	Reverse	PolyA	Poly A	GGGGCGGCCGCTTTTTTTTTTTTTTTTTTTTTTTTTTTTTT

[1] Correspond to the position in the genome of Zhangzhou isolate [24].
[2] 5'NCR; 5' non-coding region, HC-Pro; helper component-proteinase protein, P3; protein 3, 6K1; CI; cylindrical inclusion protein, 6K2; 6Kda 2 protein, VPg; genome-linked viral protein, NIa-Pro; nuclear inclusion a-proteinase protein, NIb; nuclear inclusion b protein, CP; coat protein, 3'NCR; 3' non-coding region, Poly A; Poly (A) tail.
[3] R; G+A, Y; C+T, S; G+C, M; A+C, W; A+T, K, T+G, V; A+C+G, D; A+T+G, H; A+T+C, N; A+T+G+C
GCGGCCGC; NotI restriction site

C. Narcissus late season yellows virus specific primers used for sequencing.

Primer name	Type	Position[1]	Region[2]	Sequence (5'-3')[3]
NLP1168M	Reverse	563-584	P1	TYCACCACTTGCRGTGGCTGCT
NLP190M	Reverse	606-628	P1	CCTTACTGACTTTCCGTGACCTC
NLP1332P	Forward	914-935	P1	AAATCACTCAYGGRTGGAGTGG
NLHC77M	Reverse	1156-1177	HC-Pro	CATCAAGGTTTGTCTCACACAC
NLHC161M	Reverse	1142-1163	HC-Pro	TCACAYACGTGTTCTCGATCAT
NLHC163P	Forward	1624-1645	HC-Pro	AGGAACAAGATYTCGCAGAAAG
NLHC177M	Reverse	1792-1812	HC-Pro	RCCRTTTGGGTTTGGACGCAC
NLHC71M	Reverse	1797-1818	HC-Pro	CCGTGAACCGTTTGGGTTTGGA
NLHC333P	Forward	2293-2314	HC-Pro	CCAAGAATGTTCGTAGATCATG
NLHC63M	Reverse	2445-2467	HC-Pro/P3	CCAATGGATCGCCTCCAACTAGA
NLP3334P	Forward	2749-2770	P3	GCACAACTGAGAATTTTGGAAC
NLP3160M	Reverse	3065-3086	P3	CTGCCTCTTAAATCTGCTGTGC
NLP355M	Reverse	3070-3092	P3	CTGTATCTGCCTTTCAAATCGGC
NLP3210P	Forward	3394-3415	P3	TACAAGATGTACATGATGGAGC
NLP3169P	Forward	3435-3456	P3	GGAAGGCTTYTTAGCCTACATT
NLP345P	Forward	3494-3516	P3	CAGACGAAGAGGTTGAGCACCAA
NLCI52M	Reverse	3739-3760	CI	GTGCCTGAGTTTGACTTTCATC
NLCI211M	Reverse	3739-3760	CI	GTGCYTGGGTHTGACTTTCATC
NLCI51P	Forward	4121-4142	CI	TGACAACAGGCTTCGCATTACA
NLCI274P	Forward	4552-4573	CI	AATGGMACTCCRAACAAGAAAC
NLCI143M	Reverse	4672-4695	CI	GTTRTARCACATCATTCTGTTGTC
NLCI170M	Reverse	4787-4808	CI	GGAACTTCGCTCAAACCTTTCT
NLCI220M	Reverse	4787-4808	CI	GGAACTTCGCTTAGGCCTTTCT
NLCI91P	Forward	5182-5203	CI	CACACAGAAGTTTGGGAAACGA
NLCI47M	Reverse	5350-5372	CI	GTCACTGTCTTGAAGTACTCCTG
NLCI156M	Reverse	5467-5488	CI	CCTTTGCAGCTTCCAAGACACC
POTYCI17P	Forward	5902-5924	VPg	GGGAAAGTTAGAGGAATGGGAGC
NLCI135M	Reverse	6070-6090	VPg	TWGCCCAATYTTCTCCTCTCG
NLNIA60M	Reverse	6197-6219	VPg	TTTCTTGTCGCATGATCGCAGTG
NLNIA134P	Forward	6434-6454	NIa-Pro	AYGGMGTTGGATTTGGTCCAC
NLNIA78P	Forward	6571-6593	NIa-Pro	GATCGGGATCTCATTCTTGTCAG
NLNIA162P	Forward	6586-6607	NIa-Pro	CTTGTCAGATTACCAAAGGACG
NLNIB164P	Forward	7064-7085	NIa-Pro/NIb	AAATGTCACGAGGAGGTTGGAT
NLNIB136P	Forward	7082-7102	NIb	GGATGCGTGATCAAATACATG
NLNIB110P	Forward	8014-8035	NIb	GTCGATAACACRCTAATGGTCA
POTYNIB21P	Forward	8090-8111	NIb	AYGRAATGTGYCGATTYCTTGT
POTYNIB11P	Forward	8240-8261	NIb	TRTGGTTCATGTCRCAYAAGGG
POTYNIB10M	Reverse	8242-8262	NIb	YACCCTTRTGYGACATGACCA
POTYNIB6P	Forward	8299-8320	NIb	CCTGAACGGGTTGTGTCAATTC
POTYNIB8M	Reverse	8398-8422	NIb	TCCGTATTTCACGCGTTAGCTCATC
NLCP257P	Forward	8768-8789	CP	TGCCACGACTGAARATGATAAC
NLCP93P	Forward	8842-8865	CP	CATCTHATYACATAYACACCACGA
POTYCP1P	Forward	8860-8884	CP	CCACGACAAGTGGATTTGTCGAATA
POTYCP18P	Forward	8879-8900	CP	CSAACACAAGRGCMACACAAAG
POTYCP23P	Forward	8961-8981	CP	RGCAATYYTRGAAGATTTTGA
NLCP94P	Forward	9077-9099	CP	CARTCATYGAACAYGCCAAACCA
POTYCP20P	Forward	9193-9215	CP	CTKCAGCGWAAYTTAACCGACAT

[1] Correspond to the position in the genome of Zhangzhou isolate [26].
[2] P1; first protein, HC-Pro; helper-component protease protein, P3; third protein, 6K1; 6K first protein, CI; cylindrical inclusion protein, VPg; genome-linked viral protein, NIa-Pro; nuclear inclusion a protein-proteinase protein, NIb; nuclear inclusion b protein, CP; coat protein.
[3] R; G+A, Y; C+T, S; G+C, M; A+C, W; A+T, K, T+G, V; A+C+G, D; A+T+G, H; A+T+C, N; A+T+G+C

D. Narcissus degeneration virus and potyvirus specific primers used for sequencing.

Primer name	Type	Position[1]	Region[2]	Sequence (5'-3')[3]
NDP150P	Forward	132-154	P1	AAAGCTGGGTTCACTGCTGAACA
NDP1293P	Forward	249-270	P1	TGTGGGAACCAATATAGGTCCC
NDP192M	Reverse	375-396	P1	TCCGAGCATATTCCTTTTCCAG
NDP1116M	Reverse	451-474	P1	TTTCCACAGCCACCCATGTTGTTG
NDP165P	Forward	786-807	P1	GATCGCATAAAAGGAACGAACA
NDHC72P	Forward	1371-1393	HC-Pro	GATTGCATTCGAGACGTGCCAGT
NDP1309M	Reverse	1815-1836	HC-Pro	ATGTTTGCAAAGAGCCTGTTCG
NDHC294P	Forward	1920-1941	HC-Pro	TGGGGAGACAGAGGTTACCATG
NDHC80P	Forward	2010-2032	HC-Pro	CGGAAGCATGTTAGAGGGAAGAG
NDNDP349P	Forward	2810-2832	P3	TTGTTTYAAGGAATTGATGGGGG
NDP381M	Reverse	3009-3031	P3	GTCATGTTTTGCGCCAGACACTT
NDP382P	Forward	3453-3475	P3	GCTGGTGGYGAACTCATAACTAG
NDP3295P	Forward	3555-3576	P3	GTTCTTAACTTGCTTGTACTGC
NDP3296M	Reverse	3771-3792	P3	CCGCATAGTATCTCAAATTTGG
NDCI83P	Forward	4087-4108	CI	ACTGGCGTATGCAAATGAACAC
NDCI106P	Forward	4596-4620	CI	CCAGGGAGAGAGACGGAAATATCAT
NDCI117P	Forward	5152-5175	CI	CATACGGTTTGCCAATAATGCCGC
NDCI128P	Forward	5200-5221	CI	CAAAGTGCACAAGTCAGCAAGC
NDCI84P	Forward	5364-5386	CI	CTACCAAATGCAAGCGTTGGAAG
ND6K2103P	Forward	5935-5956	6K2	TTTCCAAGGCATTGCATGATGG
NDVPG102M	Reverse	6168-6190	VPg	CTCTTGCCAGCCTTACCTTTTGA
NDVPG107P	Forward	6490-6513	VPg	AGATTGGGTTTCCAGCTCATGCAG
NDNIA85P	Forward	6624-6646	NIa-Pro	CGTAACTATAGTCCGATAGCTCG
NDNIA118P	Forward	7055-7077	NIa-Pro	GTTACCTGTAGTGAGCACCAAGG
NDNIA86P	Forward	7273-7294	NIa-Pro	TTCGTACGCTTAAGGAAGTGTC
NDNIB87P	Forward	7881-7903	NIb	AACAAGACGCGATCCTTCACAGC
NDNIB67P	Forward	8292-8313	NIb	CTTATGGTGATGTTGGCGATGC
NDNIB108M	Reverse	8471-8494	NIb	TCCGTCACCCTATTTCCAAAATCG
NDNIB1P	Forward	8596-8617	NIb	ATAGGAGCGTGGAACCAGTTAG
NDSYNIB4P	Forward	8644-8666	NIb	TGGTDGARKCGTGGGGMTAYGAT
NDNIB2M	Reverse	8746-8770	NIb	GCTGTATCTGCTAGATAAGGAGCCT
NDCP1P	Forward	9142-9166	CP	GGTACGAGAGAGTCATGCAAGCATA

[1] Correspond to the position in the genome of Zhangzhou isolate [24].
[2] P1; protein 1, HC-Pro; helper component-proteinase protein, P3; protein 3, CI; cylindrical inclusion protein, 6K2; 6Kda 2 protein, VPg; genome-linked viral protein, NIa-Pro; nuclear inclusion a-proteinase protein, NIb; nuclear inclusion b protein, CP; coat protein.
[3] R; G+A, Y; C+T, S; G+C, M; A+C, W; A+T, K, T+G, V; A+C+G, D; A+T+G, H; A+T+C, N; A+T+G+C
GCGGCCGC; NotI restriction site

E. Primers used for RT-PCR amplification and sequencing carlaviruses.

Primer	Type	Position[1]	Region[2]	Sequence (5'-3')[3]	Virus[4]
CARNCLVCP1P	Forward	7502-7523	CP	CTGACCCCAGCAATCCTTACAA	NCLV
CARNLVCP2P	Forward	7304-7325	CP	ARAARGGKTGGAGRCCTTCYTC	NeLV
CARNACP3P	Forward	7433-7454	CP	ATTGTGCAARTGCTGGTYCTTC	NCLV, NeLV
CARNCLVCP4P	Forward	7502-7523	CP	GCGGCCGCCTGACCCCAGCAATCCTTACAA	NCLV
CARNLVCP5P	Forward	7304-7325	CP	GCGGCCGCARAARGGKTGGAGRCCTTCYTC	NeLV
TU3T9M	Reverse	Poly A	Poly A	GGGGCGGCCGCTTTTTTTTTTTTTTTTTTTTTTTTTTTTT	NCLV, NeLV

[1] Correspond to the position in the genomes of narcissus common latent virus Zhangzhou isolate [74] and nerine latent virus isolate Marijiniup4 [27]
[2] CP; coat protein, Poly A; Poly (A) tail.
[3] R; G+A, Y; C+T, S; G+C, M; A+C, W; A+T, K, T+G, V; A+C+G, D; A+T+G, H; A+T+C, N; A+T+G+C
GCGGCCGC; NotI restriction site
[4] NCLV: narcissus common latent virus, NeLV: nerine latent virus

Table S3. Location of narcissus late season yellows virus and narcissus degeneration virus Japanese isolates.

Isolate	Prefecture	Location (Town or City)	Latitude	Longitude	Accession number
**Narcissus late season yellows virus (NLSYV)**					
NY-HO42	Hokkaido	Nango, Shiroishi-ku, Sapporo	43.0381	141.4124	LC664195
NY-A65	Aomori	Nanbu-cho, Sannohe-gun	40.4179	141.1924	LC664185
NY-FK266	Fukushima	Motomiya, Fukushima	37.4922	140.3401	LC664192
NY-CB1	Chiba	Tokoshiro, Kimitsu	35.2645	139.7205	LC664187
NY-CB3	Chiba	Sotominowa, Kimitsu	35.3168	139.9159	LC664188
NY-CB4	Chiba	Ezuki, Kyonan-machi, Awa-gun	35.1322	139.8563	LC664189
NY-GM252	Gunma	Fujiki, Tomioka	36.2933	138.9183	LC664193
NY-AC230	Aichi	Sannomaru, Naka-ku, Nagoya	35.1820	136.8972	LC664186
NY-FI29	Fukui	Nashigadaira, Echizen-cho, Nyu-gun	35.9823	135.9588	LC664182
NY-FI30	Fukui	Nashigadaira, Echizen-cho, Nyu-gun	35.9823	135.9588	LC664183
NY-NI263	Niigata	Kuraoka, Konan-ku, Niigata	37.8855	139.1505	LC664200
NY-HG25	Hyogo	Akurakita, Takarazuka	34.8071	135.3670	LC664194
NY-OS1	Osaka	Minowa, Toyonaka	34.7859	135.4486	LC664203
NY-HR39	Hiroshima	Sunami, Mihara	34.3676	133.0747	LC664196
NY-SM69A	Shimane	Unknown	35.1751	131.9737	LC664204
NY-YM2	Yamaguchi	Chinto, Hagi	34.4139	131.4059	LC664205
NY-KW4	Kagawa	Ikushima-cho, Takamatsu	34.3607	133.9515	LC664198
NY-M4	Miyazaki	Takaharu-cho, Nishimorokata-gun	31.9284	130.9378	LC664184
NY-F1	Fukuoka	Okawa	33.2078	130.3465	LC664190
NY-F8	Fukuoka	Kawabaru, Itoshima	33.5138	130.2656	LC664191
NY-KM9	Kumamoto	Ozu-machi, Kikuchi-gun	32.8556	130.8676	LC664197
NY-N15	Nagasaki	Uki-machi, Isahaya	32.7940	130.0778	LC664199
NY-OI4	Oita	Tanoyu-machi, Beppu	33.2783	131.4966	LC664201
NY-OI12	Oita	Fujimi-cho, Beppu	33.2866	131.4976	LC664202
NY-KG6	Kagoshima	Tenokuchi-cho, Kagoshima	31.5746	130.5283	LC664206
**Narcissus degeneration virus (NDV)**					
NY-FK266	Fukushima	Motomiya	37.5084	140.4101	LC664181
NY-AC230	Aichi	Sannomaru, Naka-ku, Nagoya	35.1878	136.9032	LC664179
NY-FI30	Fukui	Nashigadaira, Echizen-cho, Nyu-gun	35.9802	135.9624	LC664180
NY-ME229	Mie	Komei-cho, Tsu	34.7317	136.5063	LC664178
NY-KG11	Kagoshima	Uearata-cho, Kagoshima	31.5731	130.5421	LC664177

Table S4. Tentative and clear recombination sites in narcissus late season yellows virus genomes.

Isolate	Position (nt)[1]	Region[2]	Parental isolate		Recombination detection program[3]	*P*-value[4]
			Major	Minor		
NY-CB1	9015	CP	Zhangzhou (Group 5) (5')	NY-N15 (Group 1) (3')	RG**B**MCS_R	6.01×10^{-33}
NY-F1	2044	HC-Pro	NY-FI30 (Group 2) (3')	NY-GM252 (Group 4) (5')	**R**BMCS_R	1.44×10^{-30}
NY-FK266	2044	HC-Pro	NY-FI30 (Group 2) (3')	NY-GM252 (Group 4) (5')	**R**BMCS_R	5.25×10^{-30}
NY-HG25	2044	HC-Pro	NY-FI30 (Group 2) (3')	NY-GM252 (Group 4) (5')	RG**B**MCS_R	8.19×10^{-30}
NY-HR39	2044	HC-Pro	NY-FI30 (Group 2) (3')	NY-GM252 (Group 4) (5')	**R**BMCS_R	2.15×10^{-28}
NY-OS1	2044	HC-Pro	NY-FI30 (Group 2) (3')	NY-GM252 (Group 4) (5')	**R**GBMCS_R	8.01×10^{-30}
Marijiniup9	2044	HC-Pro	NY-FI30 (Group 2) (3')	NY-GM252 (Group 4) (5')	**R**GBMCS_R	1.95×10^{-34}
(JX156421)	7473	NIb	NY-OS1 (Group 2) (5')	NY-FI30 (Group 2) (3')	R**B**MCS_R	2.71×10^{-9}
	9066	CP	NY-OS1 (Group 2) (5')	NY-FI30 (Group 2) (3')	R**B**MCS_R	5.10×10^{-14}

[1] Recombination sites detected in the NLSYV genomes by the recombination detection programs, from the aligned sequences of the likely recombinant and its 'parental isolate'. Correspond to the position in the genome of Zhangzhou isolate [26] (accession number JQ326210).

[2] HC-Pro; helper component-proteinase protein, NIb; nuclear inclusion b protein, CP; coat protein.

[3] Recombinant isolates identified by the recombination detection programs: R (RDP), G (GENECONV), B (BOOTSCAN), M (MAXCHI), C (CHIMAERA) and S_R (SISCAN) programs in RDP4. The analyses were done using default settings and a Bonferroni-corrected *P*-value cut-off of 0.01in RDP4.

[4] The reported *P*-value is for the program in bold type and underlined in RDP4 and is the smallest *P*-value among the isolates calculated for the region in question. *P*-values smaller than 1.0×10^{-4} are listed.
